# Quantitative cardiovascular magnetic resonance myocardial perfusion can discriminate significant cardiac allograft vasculopathy: a multi-centre study

**DOI:** 10.1093/ehjci/jeaf201

**Published:** 2025-07-10

**Authors:** R Jablonowski, H B Andersson, C Fogarasi, H Engblom, H Arheden, P Kellman, M Carlsson, M Melin, I H Löfman, J Nickander, O Ö Braun

**Affiliations:** Clinical Physiology, Department of Clinical Sciences Lund, Lund University, Skane University Hospital, Lund, Sweden; Cardiology, Department of Clinical Sciences Lund, Lund University, Skane University Hospital, Entrégatan 7, 22242 Lund, Sweden; Department of Molecular Medicine and Surgery, Karolinska Institutet, Stockholm, Sweden; Clinical Physiology, Department of Clinical Sciences Lund, Lund University, Skane University Hospital, Lund, Sweden; Clinical Physiology, Department of Clinical Sciences Lund, Lund University, Skane University Hospital, Lund, Sweden; National Heart, Lung, and Blood Institute, NIH, Bethesda, MD, USA; Department of Molecular Medicine and Surgery, Karolinska Institutet, Stockholm, Sweden; Department of Laboratory Medicine, Karolinska Institutet, Huddinge, Sweden; Department of Medicine, Karolinska Institutet, Solna, Sweden; Department of Molecular Medicine and Surgery, Karolinska Institutet, Stockholm, Sweden; Cardiology, Department of Clinical Sciences Lund, Lund University, Skane University Hospital, Entrégatan 7, 22242 Lund, Sweden

**Keywords:** heart transplantation, cardiac allograft vasculopathy (CAV), quantitative cardiovascular magnetic resonance (CMR), myocardial perfusion

## Abstract

**Aims:**

Cardiac allograft vasculopathy (CAV) is a significant complication that contributes to both morbidity and mortality after heart transplantation. The aim of this study was to (i) assess if quantitative cardiovascular magnetic resonance (CMR) myocardial perfusion could detect different stages of CAV and (ii) establish a myocardial perfusion reserve (MPR) cut-off for significant CAV.

**Methods and results:**

Patients with a heart transplant who had performed a clinical CMR scan and invasive angiography at two centres in Sweden were included in the study (*n* = 110). Quantitative short-axis perfusion maps were acquired using single-bolus gadolinium contrast, dual-sequence perfusion imaging at rest and during stress. Global myocardial perfusion (MP) was averaged across all segments at rest and stress and MPR was defined as the ratio between stress and rest MP. All invasive angiographies were reported according to the International Heart and Lung Transplantation CAV classification. Patients were classified as follows: 53% (58/110) as CAV_0_, 38% (42/110) as CAV_1_, and 9% (10/110) as CAV_2–3_. There was a gradual decrease of stress MP and MPR with increased CAV grade. The MPR could discriminate CAV_2–3_ with an area under the curve-receiver operating characteristic of 0.88, 95% confidence interval 0.78–0.98, and using a cut-off of 2.2, the sensitivity was 100%, specificity was 68%, and positive and negative predictive values were 21 and 100%.

**Conclusion:**

In this multi-centre retrospective study, MPR assessed by CMR could discriminate CAV_2–3_ with both high sensitivity and negative predictive value and a cut-off of MPR 2.2 is suggested.

## Graphical Abstract

**Graphical Abstract:**
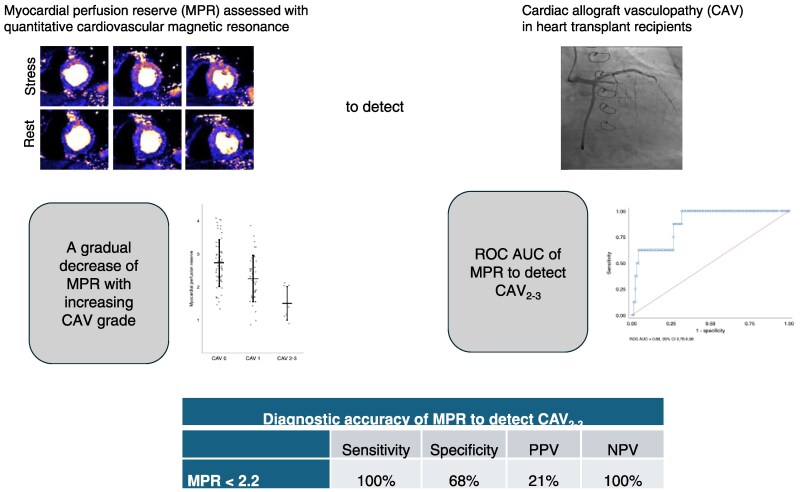

## Introduction

Cardiac allograft vasculopathy (CAV) is a significant complication that contributes to both morbidity and mortality after heart transplantation.^1^ CAV is characterized by diffuse, concentric intimal hyperplasia, leading to a general narrowing of the coronary vessels. The pathogenesis is complex, involving both immune-mediated mechanisms and classical risk factors for coronary artery disease. The prevalence of angiographically proven CAV increases with time after transplantation reaching 10% at 1 year, 44% at 10 years, and 59% at 20 years.^2^

Invasive coronary angiography using the International Heart and Lung Transplantation (ISHLT) CAV grading remains the gold standard for detecting CAV. The severity of CAV is graded into four groups according to the ISHLT criteria: CAV_0_ = not significant, CAV_1_ = mild, CAV_2_ = moderate, and CAV_3_ = severe.^3^ Increasing grades of CAV is associated with higher risk for graft loss and mortality.^2,4^ Intravascular imaging using intravascular ultrasound (IVUS) or optical coherence tomography (OCT) are more sensitive to early changes in the coronary vessels and the presence of subclinical disease. The presence of CAV according to IVUS definitions has also been linked to clinical outcomes.^5^ However, both IVUS and OCT are more invasive, associated with an increased risk of complications, and expensive compared with standard coronary angiography.

Non-invasive techniques for assessment of CAV, such as myocardial blood flow quantification using positron emission tomography (PET), demonstrate good diagnostic accuracy for CAV and have been shown to predict major adverse events.^6^ However, PET is not widely available, involves high costs, and exposes patients to radiation.^7^ Coronary computed tomography angiography (CCTA) offers a non-invasive anatomical detection of CAV but exposes patients to radiation like PET and invasive angiography, which can be considerable over time when the examinations are repeated.^7,8^ In addition, the contrast use when performing a CCTA in a population with reduced kidney function can be a limiting factor and the method cannot detect lesions in small, distal vessels.

Cardiovascular magnetic resonance (CMR) imaging using semi-quantitative myocardial perfusion (MP) and myocardial strain has previously also been linked with angiographically proven CAV.^7,9–12^ In recent years, fully quantitative CMR MP mapping has become available, which offers a standardized way for quantifying absolute MP.^13^ This technique has shown excellent accuracy compared with PET imaging in patients with atherosclerotic coronary heart disease, and decreased MP with CMR is a strong predictor of a major adverse cardiovascular event.^14,15^ CMR-derived quantitative MP was recently shown to be feasible and correlated to angiographically diagnosed CAV^16^; however, defining a myocardial perfusion reserve (MPR) cut-off for CAV would be of great clinical value.

Therefore the aims of this study were to (i) assess if quantitative CMR MP mapping could detect different stages of CAV and (ii) establish an MPR cut-off for significant CAV.

## Methods

### Study population

Consecutive patients with a heart transplant who had performed a CMR scan and invasive coronary angiography as part of their clinical follow-up at two Swedish centres (Skåne University Hospital in Lund and Karolinska University Hospital in Stockholm) between 2018 and 2023 were enrolled in the study. Major exclusion criteria included severe renal dysfunction (estimated glomerular filtration rate <30 mL/min/1.73 m^2^), contraindications to gadolinium contrast, adenosine or regadenoson, or left pacemaker leads. All coronary angiography and CMR examinations were done as surveillance and not prompted by any patient symptoms.

Patients were excluded if CMR was done due to suspicion of acute rejection or if the angiography was done more than a year prior to the CMR and showed CAV Grade 0 or 1, since progression of CAV between angiography and CMR could not be excluded in that case.

The study was approved by the regional ethics committee in Lund, Sweden (application 2004/741 with addendum 2018/948 and application 2013/319) and by the Swedish Ethical Review Authority (Dnr 2013/900, 2015/248, 2018/431, 2023-06876-01 and 2023-01479-01) and conducted in accordance with the ISHLT Ethics statement. Written informed consent was provided by all participants.

### Invasive coronary angiography

All invasive coronary angiographies were re-analysed by a cardiologist (H.B.A.) using Sectra IDS7 (Sectra Medical, Linköping, Sweden) and visually classified according to the ISHLT CAV classification: CAV_0_ = not significant, CAV_1_ = mild, CAV_2_ = moderate, and CAV_3_ = severe. The classification was done using only angiographic criteria, hence the presence of restrictive physiology and/or reduced ejection fraction was not included, and no intravascular imaging was used. Previous percutaneous coronary intervention to a segment relevant for CAV classification was regarded equal to a significant stenosis. Time from heart transplant to coronary angiography and time between CMR and coronary angiography was registered. The re-analysed results were compared with the clinical interpretation.

### CMR imaging

CMR was performed at both sites using a 1.5T Siemens Magnetom Aera or Sola MR scanner (Siemens Healthcare GmbH, Erlangen, Germany). Cine images were acquired with a steady-state free precession sequence during end-expiratory breath-hold for assessment of left ventricular (LV) mass and volumes. Quantitative short-axis perfusion maps were acquired using single bolus of gadolinium contrast [0.05 mmol/kg, Gadovist® (gadobutrol), Bayer AB, Solna, Sweden], dual-sequence perfusion imaging at rest and stress using either adenosine [140 µg/kg/min or increased to 210 µg/kg/min in the absence of adequate response to adenosine; Adenosin, Life Medical AB, Stockholm)] or regadenoson (400 µg) as vasodilator. Adenosine perfusion imaging was acquired first and resting perfusion imaging after 10 min to allow for contrast equilibrium and return to rest perfusion. When regadenoson was used, rest images were acquired before stress to avoid remaining hyperaemia, and there was 10 min between the two contrast injections.^17^ After the second contrast injection, an additional 0.05 mmol/kg contrast was injected for late gadolinium enhancement imaging acquired 10 min after the last contrast injection. Extracellular volume (ECV) maps were generated from pre- and post-contrast T1 maps and calibrated to the haematocrit.^18,19^ See Supplementary data online for typical CMR imaging parameters.

### CMR image analysis

The software Segment (Medviso, Lund, Sweden)^20^ or SyngoVia (Siemens, Erlangen, Germany) was used for LV volumetrics, which were defined by semi-automated delineation of the endocardium and epicardium in short-axis cine images. Perfusion and ECV maps were generated using the Gadgetron inline mapping software.^21^ A global average MP was calculated at rest and stress. Myocardial perfusion reserve (MPR) was calculated as the ratio of stress to rest perfusion. To correct for the rate of resting metabolism depending on haemodynamic condition the rate-pressure-product (RPP) was calculated {[resting heart rate (beats/min) × systolic blood pressure at baseline (mmHg)]/10 000], and the corrected resting perfusion and corresponding corrected MPR were calculated. Transmural endocardial-to-epicardial MP gradients were generated using the Gadgetron inline mapping software. Global ECV at rest was calculated. To evaluate diagnostic performance at the vessel level, the data set was restructured so that each patient contributed data for the three major coronary territories (left anterior descending (LAD), left circumflex (LCx), and right coronary artery (RCA)). Stress myocardial perfusion reserve (MPR) was matched to the corresponding coronary vessel,^22^ and angiographic stenosis ≥70% in a vessel supplying the myocardial region was used as the reference standard for significant coronary artery disease.

### Statistical analysis

Baseline data are presented as means and standard deviations (SDs) for normally distributed variables, medians and interquartile ranges (IQR) for skewed variables, and counts and percentages for categorical data. Multiple group comparisons were made using ANOVA with Tukey’s *post hoc* test and stepwise comparisons (where appropriate) for normally distrusted variables, Kruskal–Wallis test for non-parametric variables, and Pearson’s χ^2^ test for categorical variables. Receiver operating characteristic (ROC) curves were created, and the area under the curve (AUC) was calculated. ROC analysis was also performed to assess per-vessel diagnostic accuracy. Youden’s method with bootstrap was used to determine optimal cut-off levels for detecting CAV and per-vessel (>70% stenosis). To assess whether the predictive effect of MPR on CAV ≥2 differed between vasodilator types by performing a logistic regression model including an interaction (MPR × vasodilator). Interrater agreement of invasive angiography results were compared using kappa statistic. All analyses were performed using Stata software (version 18.0, StataCorp). All tests were two-sided, and the alpha level was set at 0.05.

## Results

### Study population

A total of 141 patients (71 in Lund and 70 in Stockholm) who had done a CMR with MP mapping after heart transplantation were identified. Out of these, seven patients did the examination due to suspicion of acute rejection, one patient did not have angiographic data available, and 23 had >365 days from angiography to CMR; thus, subsequently 31 patients were excluded from the study. Patient demographics of the remaining 110 patients are presented in *Table 1*. The median time from transplantation to CMR examination was 4.4 years (IQR 1.2–10.2 years). Most patients (69%) were males. All except three patients were treated with a calcineurin inhibitor; 87% were treated with either mycophenolate mofetil (MMF) or azathioprin, and 25% of the patients used everolimus. The majority (83%) was treated with prednisolone, and most patients were on statin and aspirin treatment (94 and 81%, respectively).

**Table 1:** Baseline characteristics of the patients

			CAV_0_	CAV_1_	CAV_2–3_	*P*-value
Number of patients		110	58	42	10	
Reason for transplantation	DCM	43 (39.1%)	24 (41%)	13 (31%)	6 (60%)	0.30
	IHD	7 (6.4%)	2 (3%)	4 (10%)	1 (10%)	
	Other	60 (54.5%)	32 (55%)	25 (60%)	3 (30%)	
Age at transplantation, median (IQR)		48 (35–56)	48 (37–52)	46 (30–56)	57 (49–59)	0.092
Sex	Male	76 (69.1%)	41 (71%)	27 (64%)	8 (80%)	0.58
	Female	34 (30.9%)	17 (29%)	15 (36%)	2 (20%)	
Weight (kg), mean (SD)		79 (15)	78 (15)	79 (16)	80 (12)	0.94
Height (cm), mean (SD)		174 (9)	175 (9)	174 (9)	174 (12)	0.89
Years from HTx to CMR, median (IQR)		4.4 (1.2–10.2)	1.4 (1.1–4.6)	7.2 (4.3–12.4)	10.9 (7.9–16.4)	<0.001
CAV grade	0	58 (52.7%)	58 (100%)	0 (0%)	0 (0%)	<0.001
	1	42 (38.2%)	0 (0%)	42 (100%)	0 (0%)	
	2–3	10 (9.1%)	0 (0%)	0 (0%)	10 (100%)	
Prior PCI			0 (0%)	0 (0%)	4 (40%)	<0.001
Tacrolimus		94 (85.5%)	54 (93%)	34 (81%)	6 (60%)	0.013
Cyclosporine		13 (11.9%)	3 (5%)	7 (17%)	3 (30%)	0.036
Everolimus		27 (24.5%)	12 (21%)	11 (26%)	4 (40%)	0.40
MMF		92 (84.4%)	50 (86%)	35 (85%)	7 (70%)	0.42
Azathioprin		4 (4.0%)	1 (2%)	2 (5%)	1 (10%)	0.43
Prednisolone		91 (82.7%)	45 (78%)	37 (88%)	9 (90%)	0.32
Statin		101 (94.4%)	53 (96%)	38 (90%)	10 (100%)	0.33
Acetylsalicylic acid		87 (80.6%)	48 (86%)	31 (74%)	8 (80%)	0.34
SBP (mmHg), mean (SD)		130 (13)	130 (13)	130 (14)	131 (13)	0.99
DBP (mmHg), mean (SD)		80 (12)	81 (11)	78 (13)	78 (11)	0.35
Creatinine (µmol/L), mean (SD)		95 (33)	90 (23)	98 (41)	114 (38)	0.069
Haemoglobin (g/L), mean (SD)		137 (18)	136 (17)	137 (20)	138 (16)	0.98
CRP (mg/L), mean (SD)		3 (4)	3 (5)	2 (2)	4 (3)	0.16
HBA1c (mmol/mol), mean (SD)		40 (10)	38 (7)	43 (13)	45 (9)	0.027
LDL (mmol/L), mean (SD)		2.1 (0.7)	2.3 (0.8)	2.0 (0.5)	1.8 (0.8)	0.064
HDL (mmol/L), mean (SD)		1.4 (0.5)	1.5 (0.5)	1.4 (0.5)	1.3 (0.4)	0.74
Triglycerides (mmol/L), mean (SD)		1.7 (0.9)	1.6 (0.7)	1.8 (1.1)	2.1 (0.8)	0.13
NT-propBNP (ng/L), mean (SD)		543 (875)	431 (808)	607 (962)	1030 (784)	0.16

*P*-value represents group comparisons between CAV grades.

HTx, heart transplantation; DCM, dilated cardiomyopathy; IHD, ischaemic heart disease; SBP, systolic blood pressure; DBP, diastolic blood pressure; CRP, c-reactive protein; HbA1c, hemoglobin A1c; LDL, low-density lipoprotein; HDL, high-density lipoprotein.

### Coronary angiograms and CAV

Coronary angiography was performed a median of 2 days (IQR −6 to 170 days) from the CMR. A total of 58 patients (53%) had CAV_0_, 42 had CAV_1_ (38%), 6 had CAV_2_ (5%) and 4 had CAV_3_ (4%). For further analysis CAV_2_ and CAV_3_ were grouped together. The agreement between the re-analysed results with the clinical interpretation was very good (kappa statistics 0.84 with standard error ±0.08). There were no discrepancies in patients with CAV_2–3_. A total of four patients had undergone percutenous coronary intervention (PCI) prior to CMR with a median of 595 days (range 286–3012 days). Two of these four patients had their PCI done 2 and 4 years prior to CMR, and the angiography closest to the CMR showed CAV > 2. One had a widespread coronary lesion and a recent PCI prior to the CMR and was deemed to be CAV2; the MPR in this specific case was 1.3. The last patient with PCI had no stress perfusion recorded and was not included in the MPR analysis.

### CMR imaging and CAV

**Figure 1:**
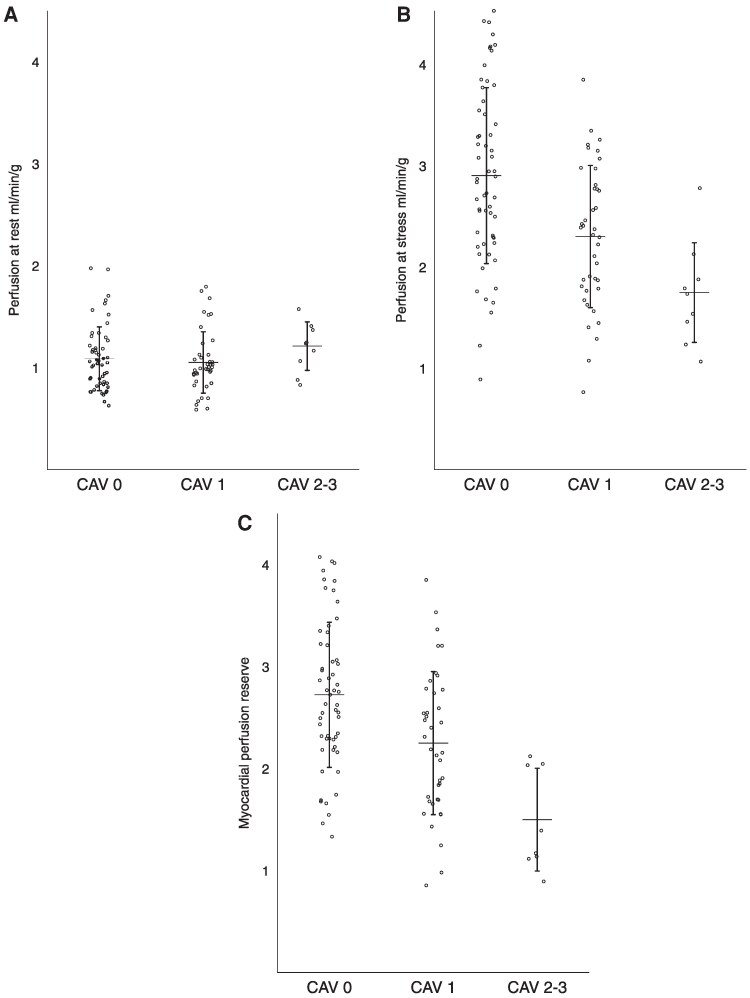
Myocardial perfusion assessed by quantitative CMR at different CAV grades at rest (*A*), stress (*B*), and myocardial perfusion reserve (MPR) (*C*).

LV size and ejection fraction were within normal limits and did not differ based on CAV grade, whereas cardiac index showed a gradual decrease with more advanced CAV grade (*Table 2*). The resting MP did not differ based on CAV severity (*Figure 1A*, Supplementary data online, *Table S1*). Stress MP showed a gradual decrease from mean 2.9 ± 0.9 mL/min/g for CAV_0_ to a mean of 1.7 ± 0.7 mL/min/g for CAV_2–3_ (*Figure 1B*, Supplementary data online, *Table S1*). The MPR also showed a gradual decrease with increasing CAV degree from a mean ± SD of 2.7 ± 0.7 mL/min/g for CAV_0_ to 1.5 ± 0.5 mL/min/g for CAV_2–3_ (*Figure 1C*, Supplementary data online, *Table S1*).

**Table 2:** Heart dimensions and function assessed by CMR

	CAV_0_	CAV_1_	CAV_2–3_	*P*-value
Number of patients	58	42	10	
LVEDV (mL), mean (SD)	144.8 (33.8)	150.6 (36.0)	125.5 (31.9)	0.12
LVESV (mL), mean (SD)	59.9 (19.7)	63.9 (21.4)	55.2 (21.6)	0.41
LVEF (%), mean (SD)	58.6 (6.4)	58.2 (8.0)	56.8 (8.2)	0.78
Cardiac index (L/min/m^2^), mean (SD)	2.9 (0.7)	2.6 (0.6)	2.2 (0.4)	0.002
Global ECV (%), mean (SD)	26.2 (4.0)	27.3 (3.8)	27.6 (1.7)	0.31

LVEDV, left ventricular end-diastolic volume; LVESV, left ventricular end-systolic volume; LVEF, left ventricular ejection fraction.

### Diagnostic performance for CMR MP vs. angiography

**Figure 2:**
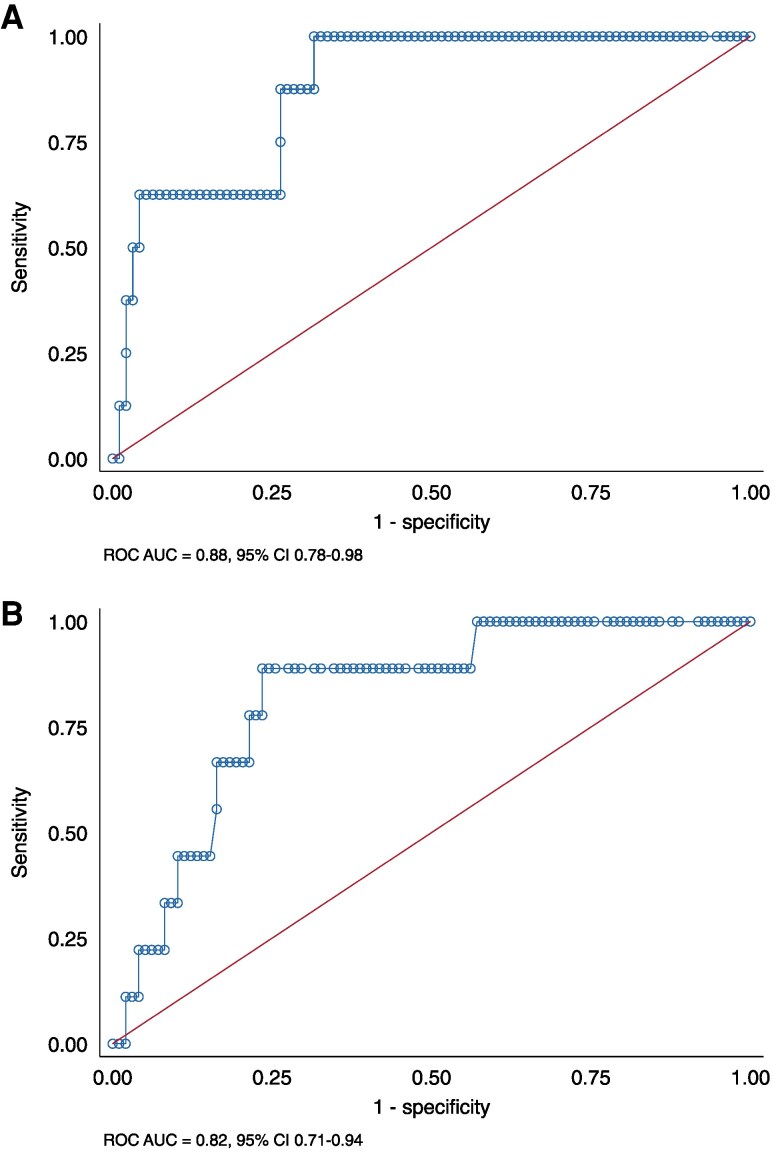
Receiver operating characteristic curves assessing the accuracy of myocardial perfusion reserve (MPR) (*A*) and MP at stress (*B*) to detect CAV_2–3_.

The MPR could discriminate CAV_2–3_ with an ROC AUC of 0.88 [95% confidence interval (CI) 0.78–0.98] (*Figure 2A*). The optimal empirical MPR cut-off (according to Youden’s method) for detecting CAV_2–3_ was 2.2 (95% CI 1.6–2.8). Sensitivity, specificity, positive (PPV), and negative predictive values (NPV) using a cut-off of MPR 2.2 are presented in *Table 3*.

**Table 3:** Diagnostic performance for different MP cut-offs

	Sensitivity (%)	Specificity (%)	PPV (%)	NPV (%)
MPR < 2.2	100	68	21	100
MP stress < 2.1 mL/min/g	89	75	24	99
cMPR < 2.2	88	63	17	98

cMPR, rate-pressure-product corrected MPR.

Using MP stress, the ROC AUC for discrimination of CAV_2–3_ was 0.82 (95% CI 0.71–0.94, *Figure 2B*), resulting in an optimal cut-off of 2.1 mL/min/g for MP stress. Sensitivity, specificity, PPV, and NPV using a cut-off of MP stress of 2.1 mL/min/g are presented in *Table 3*. The ROC AUC for discriminating a vessel with ≥70% stenosis was 0.87 (95% CI 0.79–0.96), and the vessel-level cut-off was 2.3 (95% CI 1.6–3.1).

### Perfusion corrected for RPP

Neither rest MP nor MPR differed for the whole group when corrected for RPP (uncorrected mean 1.1 ± 0.3 mL/min/g vs. corrected mean 1.1 ± 0.3 mL/min/g, *P* = 0.53; and uncorrected mean 2.4 ± 0.8 mL/min/g vs. corrected mean 2.5 ± 0.9 mL/min/g, *P* = 0.16, respectively). This was also true for patients with CAV_2–3_ (uncorrected mean rest MP 1.2 ± 0.2 mL/min/g vs. corrected rest MP 1.2 ± 0.3 mL/min/g, *P* = 0.88; and uncorrected MPR 1.5 ± 0.5 mL/min/g vs. corrected MPR 1.6 ± 0.7 mL/min/g, *P* = 0.32, respectively). Using corrected MPR, the ROC AUC for discrimination of CAV_2–3_ was 0.82 (95% CI 0.67–0.97), resulting in an optimal cut-off of 2.1 mL/min/g. One patient with CAV_2–3_ had a corrected MPR >2.2 resulting in a slightly lower sensitivity and specificity (*Table 3*).

### Comparison of adenosine vs. regadenoson stress

Adenosine was used as stress agent in 66 of the patients and regadenoson was used in 43. There was no difference in resting MP (mean 1.1 ± 0.3 mL/min/g for adenosine vs. 1.1 ± 0.3 mL/min/g for regadenoson, *P* = 0.65). However, both stress MP and MPR were higher with adenosine compared with regadenoson (stress MP mean 2.8 ± 0.9 mL/min/g for adenosine vs. 2.3 ± 0.7 mL/min/g for regadenoson, *P* < 0.01; MPR mean 2.6 ± 0.8 mL/min/g for adenosine vs. 2.2 ± 0.6 mL/min/g for regadenoson, *P* < 0.01). Both adenosine and regadenoson could discriminate CAV_2–3_ [ROC AUC 0.81 (95% CI 0.68–0.95) for adenosine vs. 0.97 (95% CI 0.94–1.0) for regadenoson]. A vasodilator-specific threshold was not determined as the interaction (MPR × vasodilator) was not statistically significant (*P* = 0.097), and the odds ratio had wide confidence intervals (95% CI 0.00004–2.3).

### Endocardial vs. epicardial MP

Across all CAV scores, the endocardial-to-epicardial MP gradient was lower during stress, and there was no difference in either rest or stress gradients based on CAV grading (see Supplementary data online, *Table S2*).

## Discussion

The current study demonstrates that automatic, fully quantitative CMR can detect different stages of CAV. Using an MPR cut-off of 2.2, the method shows high sensitivity for detecting significant disease, defined as CAV_2–3_. These findings contribute to the increasing evidence supporting the utility of CMR imaging as a robust diagnostic tool for detecting CAV and provide a potential MPR cut-off of 2.2 for clinical application.

Prior CMR studies have relied on semi-quantitative approaches, necessitating manual modifications and user-dependent analysis. While effective, these methods are inherently limited by a lack of standardization, with risk for inter-operator variability and the potential for subjective interpretation. In contrast, the fully quantitative and automated approach utilized in the current study eliminates these limitations, providing reproducible and objective results. This automation represents a key step towards standardizing CMR for clinical use in detecting CAV, ensuring consistent application across different clinical settings.

In our study, we could detect a gradual decrease of stress perfusion and the corresponding MPR with increasing CAV. This is similar to the results recently presented by Bisaccia et al.,^16^ which further strengthen the reproducibility and reliability of the method. The excellent sensitivity, specificity, PPV, and NPV achieved in this study highlight CMR’s potential as an effective screening tool for CAV. The ability to accurately detect CAV at an early stage is critical for timely therapeutic interventions, which can potentially improve graft survival and patient outcome. Our data confirm the reliability of CMR in differentiating pathological changes from normal variations, making it a viable option for routine monitoring in heart transplant recipients.

To date, there are no cut-offs proposed for fully quantitative CMR-derived MPR for detecting CAV_2–3_. Our finding of an MPR cut-off of 2.2 for significant CAV is in line with previously proposed cut-offs for microvascular disease in non-transplanted hearts of MPR <2.19.^23^ A slightly higher cut-off of 2.9 has been suggested for RPP-adjusted myocardial flow reserve using PET imaging.^24^ Correcting for RPP did not change the cut-off in our study. However, one patient changed from having an MPR <2.2 to >2.2 after correction. Given the small changes in our study, we suggest using uncorrected MPR to avoid manual analytic work during the clinical CMR evaluation. This notion is also supported by PET studies in both non-transplanted and transplanted patients suggesting that uncorrected flows provide a more accurate risk stratification.^25,26^

There was no difference in endocardial-to-epicardial MP gradient at rest or stress across CAV score 0–3. The endocardial-to-epicardial MP gradient was lower during stress for all CAV scores, with stress ratios similar to patients with structural coronary microvascular disease.^22,23^

Different vasodilators have been used as a stressor in heart transplant patients, both regadenoson^27^ and adenosine.^16^ In our multi-centre setting using both adenosine and regadenoson, we noted lower stress MP and MPR with regadenoson. Both agents were, however, able to discriminate CAV_2–3_ from CAV_0_–1.

CMR’s non-invasive nature and lack of radiation exposure are critical advantages, particularly for heart transplant recipients who are at an elevated risk of malignancy due to prolonged immunosuppression and multiple radiation exposures. By providing high-resolution imaging without radiation, CMR offers a safer alternative to other invasive diagnostic modalities like coronary angiography or CT-based imaging that includes ionizing radiation.

Beyond its application in detecting CAV, CMR’s versatility enhances its value in clinical practice. This imaging modality can simultaneously evaluate other cardiac pathologies, such as acute rejection with oedema and myocardial fibrosis, which may coexist or contribute to graft dysfunction.^28^ Additionally, its ability to precisely assess changes in cardiac function offers a comprehensive understanding of the transplant heart’s haemodynamic status, enabling a holistic approach to patient management. The span of diagnostic capability reinforces the argument for integrating CMR into routine post-transplant surveillance protocols. Thus, CMR offers a versatile tool for long-term follow-up after heart transplantation.

The findings of this study support the use of CMR as a screening tool for CAV. Moving forward, the implementation of fully automated, quantitative CMR in clinical practice could streamline the workflow and reduce the need for invasive and/or methods utilizing ionizing radiation. Furthermore, the inclusion of CMR in longitudinal studies could help elucidate the progression of CAV and the impact of therapeutic interventions over time as well prognostic implications.

## Limitations

The retrospective nature of this study introduces potential biases inherent in data collection and analysis. Retrospective studies are limited by the quality and completeness of existing clinical records, which may not fully capture all relevant variables or confounders. Consequently, the findings should be interpreted cautiously, as the lack of prospective randomization and standardization could affect the generalizability of the results. There were insufficient numbers of abnormal studies to investigate each vasodilator individually. The optimal cut-off may differ depending on which vasodilator is used, and larger studies are needed to determine whether the same cut-off can be used regardless of vasodilator type. Therefore, we did not assess a vasodilator-specific MPR cut-off for severe CAV given the small number of outcome events in our data set and the risk of overfitting. We therefore opted to use a unified MPR cut-off, which simplifies interpretation while maintaining generalizability. Also, in the current study, we did not have access to index of microcirculatory resistance (IMR) or fractional flow reserve (FFR) from invasive angiography or CCT, which could potentially increase the sensitivity for CAV detection. We did not include information on qualitative perfusion defects in the analysis as the aim of the present study was to investigate if quantitative perfusion alone could detect different stages of CAV. Furthermore, we included four patients with prior PCI, which could potentially affect our results, but since PCI was performed on average 595 days before CMR, these subjects were included in our final analysis.

The study was conducted at only two centres, which may limit its external validity. Differences in imaging protocols, patient demographics, and clinical practices between institutions could influence the performance of CMR in broader clinical settings. A prospective, multi-centre approach involving diverse populations and clinical workflows would strengthen the evidence for the widespread applicability of the proposed automated CMR method. However, many studies in the field are based on single-centre studies and the fact that two centres were included strengthens the results and conclusions from the present study considerably.

The study cohort included a limited number of patients with severe CAV, which could impact the accuracy of the performance metrics (sensitivity, specificity, PPV, and NPV) for detecting advanced disease. This underrepresentation may result in overestimation or underestimation of the method’s diagnostic capability in severe cases. We did also not evaluate the association between MPR cut-offs and adverse outcomes given the limited follow-up times after CMR exams. Future studies with larger cohorts and longitudinal follow-up that encompass a full spectrum of disease severity are necessary to validate the findings and determine the robustness of CMR in detecting severe CAV.

In conclusion, the current multi-centre study demonstrates that inline, fully quantitative CMR can detect different stages of CAV. Using an MPR cut-off of 2.2, this method shows high sensitivity for detecting significant vasculopathy, defined as CAV_2–3_. Further studies, including larger patient cohorts, stratified on vasodilator type, additional centres, and longitudinal follow-up, are needed to validate the finding and to better evaluate the role of CMR in detecting CAV after heart transplantation.

## Supplementary Material

jeaf201_Supplementary_Data

## Data Availability

The data underlying this article will be shared on reasonable request to the corresponding author.
