# Supplementary material for: Quantitative cardiovascular magnetic resonance myocardial perfusion can discriminate significant cardiac allograft vasculopathy: a multi-centre study
Source: Eur Heart J Cardiovasc Imaging. 2025 Jul 10;26(10):1623–30. doi: 10.1093/ehjci/jeaf201 (PMC12481015; doi:10.1093/ehjci/jeaf201)
Supplement: jeaf201_Supplementary_Data [file jeaf201_supplementary_data.docx]

**Supplements**

**CMR parameters**

*Cine imaging:* Balanced steady state free precession (bSSFP) cine imaging was acquired with the following typical imaging parameters: repetition time (TR) = 2.7 ms, echo time (TE) = 1.2 ms, flip angle 60°, spatial resolution 1.5 × 1.5 × 8 mm with no slice gap and field of view (FOV) 270 × 320 mm^2^.

*Perfusion imaging*: Typical imaging parameters were: bSSFP single shot readout, TE 1.0 ms, TR 2.5 ms, flip angle 50°, FOV 360 × 270 mm^2^, slice thickness 8.0 mm, parallel acquisition technique factor 3, acquisition time per single shot slice 142 ms and saturation delay 105 ms.

*Extracellular volume (ECV) imaging:* Pre-contrast T1 maps were acquired using a Modified Look-Locker inversion recovery (MOLLI) 5s(3s)3s prototype sequence with typical imaging parameters: single shot SSFP in end-diastole, flip angle 35°, pixel size 1.4 × 1.9 mm^2^, slice thickness 8.0 mm, imaging duration 167 ms, TE/TR 1.12/2.7 ms, matrix size 256 × 144 and FOV 360 × 270 mm^2^. Post-contrast T1 maps were acquired with the same slice position as the pre-contrast T1 maps. ECV maps were generated from pre and post-contrast T1 maps, calibrated by hematocrit.

**Table S1.**

|  | CAV0 | CAV1 | CAV2-3 | p-value ANOVA | p-value CAV0 vs. CAV1 | p-value CAV1 vs. CAV2-3 |
| --- | --- | --- | --- | --- | --- | --- |
| Rest MP | 1.1 (0.3) | 1.1 (0.3) | 1.2 (0.2) | 0.36 | 0.83 | 0.33 |
| Stress MP | 2.9 (0.9) | 2.3 (0.7) | 1.7 (0.5) | <0.001 | <0.01 | 0.14 |
| MPR | 2.7 (0.7) | 2.2 (0.7) | 1.5 (0.5) | <0.001 | <0.01 | 0.02 |

MP denotes myocardial perfusion, MPR myocardial perfusion ratio.

**Table S2.**

|  | CAV0 | CAV1 | CAV2-3 | p-value |
| --- | --- | --- | --- | --- |
| endo/epi-ratio rest MP | 1.1 (0.1) | 1.1 (0.1) | 1.1 (0.1) | 0.66 |
| endo/epi-ratio stress MP | 0.9 (0.1) | 0.9 (0.1) | 0.9 (0.1) | 0.93 |

MP denotes myocardial perfusion.
